# Social and cultural determinants of antibiotics prescriptions: analysis from a public community health centre in North India

**DOI:** 10.3389/fphar.2024.1277628

**Published:** 2024-01-25

**Authors:** Arunima Mukherjee, Rashmi Surial, Sundeep Sahay, Yogita Thakral, Amandeep Gondara

**Affiliations:** ^1^ Department of Informatics, University of Oslo, Oslo, Norway; ^2^ Society of Health Information Systems Programmes, New Delhi, India; ^3^ Centre for Sustainable Healthcare Education (SHE), Faculty of Medicine, University of Oslo, Oslo, Norway

**Keywords:** antimicrobial resistance, prescription practices, drug quality, antibiotic prescription, culture sensitivity testing, branded antibiotics

## Abstract

This paper explores the socio cultural and institutional determinants of irresponsible prescription and use of antibiotics which has implications for the rise and spread of antimicrobial resistance (AMR). This study describes the patterns of prescription of antibiotics in a public facility in India and identifies the underlying institutional, cultural and social determinants driving the irresponsible use of antibiotics. The analysis is based on an empirical investigation of patients’ prescriptions that reach the in-house pharmacy following an outpatient department (OPD) encounter with the clinician. The prescription analysis describes the factors associated with use of broad-spectrum antibiotics, and a high percentage of prescriptions for dental outpatient department prescribed as a precautionary measure. This paper further highlights the need for future research insights in combining socio-cultural approach with medical rationalities, to further explore questions our analysis highlights like higher antibiotic prescription, etc., Along with the recommendations for further research.

## 1 Introduction

Irresponsible use of antibiotics, including overuse and misuse, is one of the biggest drivers of Antimicrobial Resistance (AMR) (Llor and Bjerrum, 2014). AMR occurs when bacteria, viruses, fungi, and parasites mutate over time and stop responding to previously prescribed antibiotics, making it difficult to treat infections, thereby, increasing the risk of disease spread, severe illness and, even, in certain cases, death (van Saene et al., 1998; Hulscher et al., 2010).

The issue of irresponsible antibiotic use is particularly crucial, especially in low- and middle-income countries (LMICs) struggling with the huge existing burden of infectious diseases. Within such resource constrained contexts, there exist both formal and informal pathways through which individuals can access antibiotics. Formally, patients receive prescriptions from physicians, and they subsequently visit pharmacies to purchase antibiotics. Informally, antibiotics are available over the counter (OTC) without the need for a prescription. The choice of accessing antibiotics through either formal or informal pathways is influenced by various social and cultural factors, including income levels, access to healthcare professionals, advice from friends and family, and other considerations. Patients have the option to either choose the formal pathway and consult a physician for a prescription or directly approach a pharmacist through an informal pathway to purchase antibiotics without a prescription. Additionally, individuals may engage in self-medication or seek guidance from friends and family, which influences the pattern of self medication among patients. In our analysis, we focus on prescription as the unit of study, recognizing its historical significance in shaping medical practices and structuring the clinicians-patient relationship. In LMICs, the prescription also plays a pivotal role in shaping the patient’s interaction with the pharmacist. Beyond dispensing drugs, pharmacists provide essential information to patients on how to properly consume the prescribed medications (Green et al., 2023). These interactions contribute to the formation of relationships that influence access to therapeutic treatments and facilitate nuanced examinations of issues related to medical power, the politics of therapeutic authority, and the intricate connection between knowledge and practice (Greene and Watkins, 2012).

This paper explores the different dimensions of prescription, including the pattern of prescription of antibiotics, clinical diagnosis among patients with different demographics. The approach taken in this paper is to determine the social, cultural and institutional conditions that shape prescribing practices, while acknowledging this is a complex process shaped by multiple interconnected conditions. We thus approach prescriptions not only based on medical rationalities, but how contextual conditions shape the creation of an “alternative rationality” in how prescriptions are interpreted and used. This leads to the formulation of the research question: “*how do social and cultural factors shape antibiotic prescriptions and create compliance challenges with formal guidelines on antibiotic use?*” Such an analysis has relevance for both theory and practice. For theory, we contribute by situating the study of antibiotic prescribing to a broader contextual analysis, which provides for a more nuanced understanding of not only the patterns of prescriptions, but also the underlying reasons of why such patterns take place. This cautions against putting the onus of “non-responsible” antibiotic consumption only on the individual and sees these patterns to represent an “alternative rationally” that is socially, culturally and institutionally shaped. For practice, our analysis can provide guidance on the underlying prescription patterns, the points of divergence from specified antibiotic use guidelines, which can inform antibiotic stewardship.

## 2 Social, cultural and institutional factors influencing antibiotic use

### 2.1 Global context

Antibiotics prescribing presents a crucial contemporary paradox for healthcare provision. On one hand, unnecessary use of the newest broad-spectrum antibiotics when in some cases narrow-spectrum and older agents would suffice, which can lead to resistance, harm patients and increase treatment costs. On the other hand, unjustified therapy with narrow spectrum antibiotics that may or may not be effective in treating infections can also be detrimental to patients (Liu et al., 2019). Excessive use of antibiotics not only jeopardizes the ability to treat and prevent microbial infections, but also increases the risk of infections in routine medical procedures like C-sections and dialysis. To address the crucial challenge of “no action today, no cure tomorrow”, is to more responsibly approach prescribing decisions, which is sensitive to this paradox and its implications. This represents a non-trivial challenge since antibiotics prescribing is a complex process, influenced by multiple sociocultural, economic and institutional drivers. Charani (2022) making the argument that “culture matters” notes different and interacting socio-cultural, behavioral and organizational conditions influencing antibiotics prescribing. Cultural conditions emerge from aspects of medical specialisms, national cultural characteristics, behavioral traits and organizational policies and practices, which play out at multiple levels of societies, hospitals and individuals (Touboul-Lundgren et al., 2015). At the level of hospitals, Liu et al. (2019) described the adoption of the Teixeira Antibiotics Prescribing Behavioral Model (TAPBM) to understand the multiple interconnections between intrinsic and extrinsic factors that shape issues such as the percentage of prescriptions containing antibiotics and the multiple antibiotics prescribed. At the individual level, the authors noted that female clinicians were less likely to prescribe antibiotics as they were more comfortable in adopting the “wait and see” principle, while also being more likely to succumb to requests by patients to prescribe antibiotics.

At the national level, countries publish country reports annually on antibiotics prescription statistics, for example, Cars et al. (2001) studied prescriptions across European countries. While Holland showed the lowest use of antibiotics, France was about 4 times higher, Belgium and Italy 3 times and Germany 1.5 times. The median in Europe was 2.1 defined daily uses per 1,000 population per day, in Norway and Sweden was 1.3 and 3.9 in Finland and France. They related these differences to the different cultural ideas which people in these countries held about health, the causes and labeling of diseases, coping strategies and treatment modalities (Cars et al., 2001). Similarly, Deschepper et al. (2008) analyzed reasons underlying such cultural variations across 14 European countries, which have implications on antibiotic prescribing. Protestant societies, with their values of austerity and simplicity, tended to favour limited medication including antibiotics, while Catholic societies, which were more hierarchical and ritualistic, favoured higher levels of antibiotics use (Zhang et al., 2019). Deschepper et al. (2008) identified how positive policies of government funding and reimbursements contributed to fewer use of generic drugs and greater use of newer and broad-spectrum antibiotics in France as compared to Germany. If healthcare is better funded and drug use reimbursed, more broad-spectrum drugs be seen to be used in Belgium (Deschepper et al., 2008). In China, there was a clear correlation between lower levels of household income with higher levels of antibiotic prescriptions (Liu et al., 2019). This may perhaps be connected to the hierarchy issue, that when people feel more vulnerable, they seek control with medicines. Liu et al. (2019) argued that the over-prescription of antibiotics in China has its roots in the distorted pricing system shaped by the transition of the country to a market system, with inadequate government funding, fee for service payments, and the 15% profit margin on the sale of medicines, representing 45% of hospital revenues. Behavioral aspects also play an important role in shaping practices of antibiotic prescribing (Liu et al., 2019; Charani, 2022).

### 2.2 The indian context

In India, there have been multiple studies relating to prescriptions containing single antibiotics *versus* poly-pharmacy, differences in prescription patterns between males and females across age groups and locations (rural/urban), indiscriminate use of antibiotics, compliance of prescriptions with Essential Drug Lists (Srishyla et al., 1994; Bhagavathula et al., 2021).

Over the years, the Government of India has introduced various policies, such as the Policy for Containment of AMR (2011) to prevent over the counter (OTC) sales of antibiotics. The Central Drugs Standard Control Organization released in 2014 the Schedule H1 drug list consisting of 24 antibiotics, making it mandatory for pharmacists to maintain a record of the sales of these drugs. These drugs are sold with the symbol Rx, in red colour with the caution “*SCHEDULE H1 PRESCRIPTION DRUG–CAUTION. It is dangerous to take this preparation except in accordance with medical advice. Not to be sold by retail without the prescription of a Registered Medical Practitioner*” (Agarwal and Chairman, 2017). In 2016, the “Redline campaign” was launched by the Ministry of Health and Family Welfare (MoHFW) along with the Organization of Pharmaceutical Producers of India to create public awareness of the rational use of antibiotics. A vertical red line on the antibiotics packaging as a message for the dispensing pharmacist to dispense only with a supporting prescription. The campaign was launched with several key objectives: learning how to identify prescription drugs, curbing self-medication and becoming more aware of the dangers of misusing antibiotics (Travasso, 2016).

As India transitions its health financing system, government investment in health insurance has expanded, with a strong focus on expanding the coverage of services to the poor. The reasonable use of antibiotics prescriptions needs to be thoroughly scrutinized for the best interests of patients (Gebeyehu et al., 2015). India is the third-largest producer of generic drugs worldwide, with generic medications being 80%–85% less expensive than brand-name medications. The government has issued the National List of Essential Medicines (NLEM) based on criteria of safety, efficacy, and cost-effectiveness, which the clinicians and hospitals are expected to promote through their prescriptions. There is a significant gap in data on prescription patterns among private facilities and tertiary teaching hospitals, as the majority of research has focused on primary and secondary level government hospitals (Venkatesh et al., 2011).

Influencing prescription practices are multiple socio-cultural factors. A qualitative study conducted in the villages in districts of Haryana state in India, identified that none of the participants were correctly able to define the term antibiotics. Participants were more likely to buy drugs without a prescription directly from a pharmacy if they had trouble getting an appointment with an allopathy clinicians, whether for logistical or financial reasons (Tripathy et al., 2018). Other factors that encourage self-medication included limited health awareness and education and related to antibiotics was also seen amongst the educated middle-class population in India (Mugada et al., 2021).

The WHO (World Health Organization) in 2017 developed the **AWaRe** Classification of Antibiotics to support antibiotic stewardship efforts, including groups of Access, Watch and Reserve. **Access** group includes antibiotics that have activity against a wide range of commonly encountered susceptible pathogens while also showing lower resistance potential. The **Watch** group includes antibiotics that have higher resistance potential. The **Reserve** group includes antibiotics that should be reserved for treatment of confirmed or suspected infections due to multi-drug-resistant organisms (Mugada et al., 2021).

Two national level guidelines from the National Center for Disease Control (NCDC), Indian Council of Medical Research (ICMR) have developed recommended treatments for common infectious diseases and the use of antibiotics, based on contemporary scientific evidence. The WHO has also recommended Standard Treatment Guidelines (STGs) to improve patient care while enhancing cost savings. However, local patterns of resistance often differ across geographical regions and WHO recommends that STGs should be based on local antibiograms, derived from syndromes and diseases, and should specify the type of clinical setting (such as Outpatient/Inpatient departments of ICUs) (Venkatesh et al., 2011). The ICMR has also developed evidence-based treatment guidelines for treatment of common syndromes in 2017 to rationalize the usage of antibiotics based on the National List of Essential Medicines (NLEM) and the need to prescribe antimicrobials only when needed in treating an infection with clear diagnosis. Some key aspects of the national guidelines, relevant for our analysis is summarized in Table 1 below (Llor and Bjerrum, 2014).

**TABLE 1:** Key features of national antibiotics prescribing guidelines.

S.No.	ICMR guidelines	NCDC guidelines
1	Make clinical diagnosis before starting treatment	Send the patient for follow up on standard investigations to make correct diagnosis
2	Limiting empirical treatment of antibiotic therapy	Antibiotic should be started only after sending culture if facilities are available
3	Knowing your bug before starting antibiotic treatment	Assessing the factors affecting activity of antimicrobials
4	Choosing appropriate antibiotics for treatment and modifying them depending on culture sensitive reports	Review antibiotic therapy and it should be escalated or de-escalated accordingly after receipt of culture report

After the introduction, where we have outlined the motivation for this paper and the research question along with a brief overview of relevant literature concerning social, cultural and institutional factors influencing antibiotic prescribing, we have describe the methods, and then the findings and results. The results are then interpreted, and conclusions are drawn.

## 3 Methods

### 3.1 Study site

This study was conducted at a 30-bed public community health centre, providing health services to a catchment population of 12,900, comprising 33% urban and 66% rural (Chandramouli and General, 2011), approximately equally distributed between males and females, with children comprising about 8.5% of the population.

The community health centre under study provides outpatient facilities, including for medicine, microbiology, obstetrics and gynaecology, ophthalmology, pediatric, dental and skin. The facility also has a microbiology lab, established in 2021, where urine, stool and pus culture samples are tested for Antimicrobial Sensitivity Tests (AST). The facility caters to a daily outpatient load of 200–250, and hosts two pharmacies (a dispensary and a civil pharmacy). The dispensary is located inside the hospital premises, open from 9 a.m. to 4p.m., providing free notified essential drugs. The civil pharmacy, located outside the premises of the health facility, provides 24/7 services and a 10%–15% discount on drugs. This pharmacy lies alongside multiple private pharmacies, where patients buy drugs both for particular reasons of perceived quality and availability.

### 3.2 Study design and data collection approaches

The study is designed within the framework of a larger 4-year research project, Equity AMR. This project explores the issues of inequities associated with AMR across three dimensions; prescription, diagnostics and surveillance. Inspired by systems thinking, the study examines how these interconnected dimensions influence AMR within largely rural public health settings in northern India. This paper uses a mixed method approach to explore the prescription dimension. We adopt a mixed methods approach. The timeline for data collection is August-December 2022. This is an ongoing research to study the prescriptions over 1 year period across 3 facilities in the region to gain understanding of seasonal, cultural and medical variations influencing prescribing behaviour. For this paper, we present results from one of the facilities among three facilities with the initial data collected over a period of 5 months. The data collection tools are described below.

### 3.3 Data collection methods

#### 3.3.1 Structured questionnaires

Structured questionnaires were developed to collect relevant information from patient’s prescription when patient went to purchase medicines from the in house-pharmacy of the hospital. One researcher would stand at the hospital pharmacy in the health facility and randomly select patients’ prescriptions. At a time, the researcher covered only one pharmacy. Regardless of the dosage form or patient’s age, all prescriptions for antibiotics were eligible for inclusion. A simple random sampling method was used and only those prescriptions were selected which had one or more antibiotics included or were related to infections which potentially would involve the use of antibiotics in the future.

About 100 patients would visit the pharmacy every day, and the researcher, who was a microbiologist by training, would go to the queue where the patients were standing, and take consent from the patient to look at their prescriptions, and about 10 of them would be selected randomly from that queue, whose prescriptions had antibiotics prescribed. The phone camera was used to take a picture of the prescriptions. After blocking out the patient details (like names, they were not taken into excel), the rest of the data in the prescriptions that contained data on demographics (age, gender, location, slip owner), other details such as symptoms of illness (diagnosis in some cases), drug names (antibiotics and others), dose and duration of treatment, and legibility of the prescriptions was noted in excel. The tool used based on these above parameters, was first piloted based on 30 prescriptions, and then revised. Data collection was then initiated with the revised tool. Data from the prescriptions was then taken into an Excel sheet, from where it was transferred into a database to facilitate easy analysis and the generation of analytical dashboards.

Inclusion criteria: Any patient who has been prescribed antibiotics.

Exclusion criteria: Patients with a diagnosis of hypertension, diabetes or heart disease were excluded.

#### 3.3.2 Semi-structured interviews with clinicians, pharmacists and patients

While the data from the prescription gave a quantitative view of the prescription patterns, to try and understand better, the why behind these patterns, we conducted semi-structured interviews with a few patients, clinicians to understand broadly when do they feel the need to prescribe antibiotics and for what conditions (from OPD, which included different OPD specialties–General Practitioners, Dental, Microbiologist, Dermatologist and Anaesthetist) and the two pharmacists. While capturing patient prescriptions, a few patients were randomly selected to ask if the patient knew about the type of medicine that was being prescribed and the treatment plan. Clinicians were interviewed to discuss with them their prescription practices, why they felt the need to prescribe antibiotics, for what kind of infections, which antibiotics do they think to be most effective and why? For follow up, we took permission from some patients who visited the microbiology lab for AST testing and who had been prescribed antibiotics and obtained their phone numbers with due permission. We note the duration of the antibiotics dose and would call them to understand how well they complied with or not with their prescriptions. Short vignettes were prepared, to develop from the patient side, an understanding of their use of the prescriptions.

#### 3.3.3 Observation of patient–pharmacist interactions

While capturing patients’ prescriptions from the pharmacy, patient-pharmacist interactions were also observed to understand how the pharmacist explained the prescription to the patient and what kinds of questions and clarifications the patients asked with respect to the medicines being prescribed.

### 3.4 Data analysis

Multiple modes of data analysis were used and also the quantitative data was triangulated with the qualitative data being collected through the interviews and observations. The prescriptions were obtained, which was entered into Excel and was exported to DHIS (District Health information system) for analysis. Some of the core indicators analyzed are: demographics, average number of drugs per prescription, % of drugs per prescription, % of prescriptions with single and multiple antibiotics, commonly prescribed antibiotics against symptoms, etc.

We then looked at the transcripts from the interviews and observations, and in some cases tried to triangulate that with other pieces of data. The vignettes from the patient follow ups gave us a sense of the perspective of patients. Taken together, these different modes of data analysis helped to make inferences about how prescription practices were, and how they were influenced by social, cultural and institutional factors. We subsequently also discussed our inferences with medical experts and pharmacologists, to build a better and more nuanced understanding of the issues.

### 3.5 Ethical consideration

The study protocol received approval from the Health Department where the health facility was based. All patients and clinicians’ identities were removed, and patient data was handled with the utmost confidentiality.

## 4 Results

### 4.1 Demographic details of patients studied

Out of the prescriptions studied, 290 (43%) were males and 391 (57%) females, indicating females were the higher recipient of antibiotics. The age group distribution indicated 16.59% of patients were below 5 years, 22% between 5–14 years and 61.38% above 14 years 62.4% of the patients were from rural areas and the rest were from urban areas. Summary demographic characteristics of patients are summarized in Table 2.

**TABLE 2:** Demographic details of the prescriptions.

S No.	Demographic categories	Number (n = 681)	%
1	**Gender**		
	Male	290	43
	Female	391	57
**2**	**Age**		
	0–5 years	113	16.59
	5–14 years	150	22
	Above 14 years	418	61.38
**3**	**Area**		
	Rural	424	62.4
	Urban	257	37.6
**4**	**Owner of slip**		
	Self	436	64
	Others	245	36

Others: the patient did not come to take his medicines, he/she sent an attendant and, in the case of children, the parents took the medicine.

Self: the patient came to take the medicine by himself.

### 4.2 Characteristics of antibiotic prescribed

From the 681 antibiotic prescriptions captured, of which 97% of prescriptions included antibiotics and 2.05% of prescriptions did not. We found that 98.8% of the drugs were prescribed from the Essential Drug List. On an average, 2.6 drugs were included per slip and 87.2% of drugs prescribed were by their generic names. Diagnosis/symptoms were mentioned in 52% of the prescriptions and the duration of illness was mentioned in only 16% of the prescriptions. 92.5% of the prescriptions were legible, which means that they were easily understood by researcher, i.e., prescription with a clear handwriting was considered to be a legible prescription. A summary of prescription patterns is described in Table 3.

**TABLE 3:** Key characteristics of the antibiotic prescriptions.

S. No.	Indicator	Number (N)	%
1	Total prescriptions	681	
2	Total antibiotic prescription	667	97.9
3	Total prescription without antibiotics	14	2.05
4	Drugs from EDL list	673	98.8
5	Drugs by generic name	594	87.2
6	Legibility of prescription	629	92.5
7	Prescriptions mentioned with diagnosis	360	52
8	The duration of illness was mentioned in	108	16

### 4.3 OPD wise antibiotic prescriptions

We found that 27% of the antibiotics were from the Dental OPD, followed by 20% from Paediatrics, 19.3% from General Medicine, and 11% from Dermatology OPD. OPD wise break up is summarized in Table 4. In standard practice, non clinical departments like microbiology and pathology do not prescribe drugs. However, as this study is based at a district hospital in a rural area where physicians come for their mandatory rural services as part of their medical degree. The mandatory rural service binds physicians with conditions to serve in their respective states for a certain fixed period, in rural areas. So, these physicians provide patient consultations and write prescriptions irrespective of the discipline they graduate in. So, in this case, some of the prescriptions are written in pathology and microbiology departments.

**TABLE 4:** Summary of OPD wise breakup of antibiotics prescriptions.

S. No.	OPD	Percentage (%)
1	Dental	27.11
2	Paediatrics	20.30
3	General Medicine	19.3
4	Dermatology	10.90
5	Eye	7.40
6	Pathology	4.50
7	Gynaecology	3.50
8	Microbiology	2.90

### 4.4 Patterns of antibiotics prescribed

The top five antibiotics prescribed among the WHO AWaRe, their class and spectrum of action, numbers and percentage are summarized in Table 5 below.

**TABLE 5:** Patterns of antibiotic prescriptions as per WHO AWaRe classification.

S. No.	Type of antibiotic prescribed (access group)	Class of antibiotic	Broad or narrow spectrum	No. Of prescriptions (N = 681)	%
1	Amoxyclav	Aminopenicillins + Beta lactamase inhibitor	Broad spectrum	177	26.53
2	Metronidazole	Imidazoles	Narrow spectrum	92	13.79
3	Amoxicillin	Aminopenicillins	Narrow spectrum	64	9.59
4	Doxycycline	Tetracycline	Broad spectrum	55	8.32
5	Nitrofurantoin	Nitrofuran	Narrow spectrum	27	4.04
	(Watch group)				
6	Azithromycin	Macrolides	Broad Spectrum	88	13.19
7	Ciprofloxacin	Fluoroquinolones	Broad spectrum	73	10.94
8	Ofloxacin–ornidazole	Fluoroquinolones	Broad spectrum	57	8.54
9	Cefixime	3rd generation Cephalosporins	Broad spectrum	33	4.94

A total of 9 different types of antibiotics were prescribed in different conditions. Out of these, Amoxyclav, Metronidazole, Amoxicillin, Doxycycline and Nitrofurantoin belonged to the Access category. While Cefixime, Azithromycin, Ciprofloxacin, Ofloxacin- ornidazole belonged to the Watch group category. No antibiotics from the Reserve group were prescribed.

### 4.5 Quality of drugs available in the hospital

The child specialist in the hospital tended to prescribe branded medicines which are available in the civil pharmacy, since they believed that the quality of medicines available in the hospital dispensary was not as good as compared to the medicines available privately. To illustrate, the specialist asked the researchers to taste Vitamin C tablets available in the hospital dispensary and compare them with the ones available outside. He explained that “if the taste and quality of such a basic thing is poor inside the hospital then what can we expect from the other medicines”. The physician was hesitant to share further details about the drug quality and pointed towards the political pressures involved in the tendering of drugs in the hospital, and how these affected the quality of drugs purchased by the hospital.

### 4.6 Levels of awareness about antibiotics in patients

Moreover, on interviewing the parents of the children, they also told that they ask clinicians to prescribe branded medicines as they see them to be good in quality and do not want to take any risk for their children’s health. Parents mentioned that “they can be careless in their own case and adults in their family and prefer to buy medicine from a pharmacy for 1 or 2 days of the initiation of the health problem, such as for fever, cough, diarrhoea, but not in the case of children. For children, we would rush to the hospital, as soon as we get an idea that a child is not feeling well”. In total, 71 prescriptions from Paediatrics OPD included branded medicines (medicines prescribed with the brand name). For example, for Cefixime, they prescribed Cefolac. A key reason for the pediatric OPD for prescribing branded medicines was also the perception of parents that drug quality in the hospital pharmacy was poor, and they needed to then buy branded drugs from external pharmacies.

Analysis of interview transcripts with 16 patients, helped to identify relevant themes, centred largely around issues of awareness. Only one male patient aged about 50 years and 2 females who had heard about antibiotics but not much more, and believed the clinicians need to know about the consequences. Clinicians should know about antibiotics and not them. One female patient who was asked why she had been prescribed antibiotics for a long-term pain in her teeth, said she was unaware of how antibiotics could help. This response was similar to the reply by another female who had been prescribed antibiotics for her child. There was another male patient who had a fever and was prescribed antibiotics. When asked, he said he knew he had some medicines for fever but had no idea of the specific role of antibiotics. There was another female patient, in her mid-twenties, who we observed buying antibiotics from the pharmacist. She said she had heard about antibiotics, since the clinician, who was a prior acquaintance of hers, had informed her about why he was prescribing her antibiotics to be taken for 5 days. We found two male patients, aged about 35 and 50 years who were diagnosed with Upper Respiratory Tract Infection, but had not been prescribed with antibiotics, much to their dismay and disappointment, since they believed that without antibiotics the fever would not go away. Patients who had previously been prescribed with antibiotics for a particular health problem, built expectations that for future health episodes of all kinds, they must be given antibiotics. The other 8 patients refused that they had no idea about what are antibiotics.

The majority of the clinicians responded that they do counsel patients on the use of antibiotics, except one clinician who said that since they did not have much time, and it is the pharmacist who explains the dosage, duration and side effects to the patients. We estimated that only 20% of the patients who were asked to come for follow up, actually came. The pharmacist believed that he was the only one who explained to patients about the administration of antibiotics, but not about the drugs themselves, which they thought should be done by the clinicians. Whether the patients knew about the drug or not, they would give some information to build the satisfaction of patients and but would direct them to the clinicians for more information. But the clinicians never told them about what medicines were prescribed and patients saw them being unapproachable and felt fear in talking to them. From our own observations, we hardly ever see clinicians counselling patients on the use of antibiotics, and the only information to patients is from the pharmacist, but primarily related to the administration of the medicines.

### 4.7 Compliance of prescriptions with national guidelines

We made some inferences on how we saw the prescribing practices to complying or not with the national guidelines. Our summary analysis is presented in Table 6 below.

**TABLE 6:** Compliance of prescriptions with national guidelines.

ICMR guideline	NCDC guidelines	Levels of compliance based on data
Make a clinical diagnosis before starting any treatment	Send the patient for follow up on standard investigation for correct diagnosis	The clinical diagnosis (presumptive or confirmatory) was made in 52% of the prescriptions and the rest, 48% were given antibiotic treatment based on signs and symptoms
Limiting empirical treatment of antibiotic therapy	Antibiotic should be started only after sending appropriate culture if facilities are available	Out of 681 prescriptions, 93 patient prescriptions were sent for urine and 25 for pus culture. All patients who were advised for pus culture were started with empirical treatment of Amoxyclav. Out of 93 patient prescriptions advised with urine culture, 77 of them were prescribed empirical treatment with Nitrofurantoin (for adults) or Cefixime (in case of Pediatrics). Only in 16 of the patient prescriptions clinicians wait for the AST report
Knowing your bug before starting antibiotic treatment	Assessing the factors affecting activity of antimicrobials	ASTs rarely conducted before empirical therapy
Choosing appropriate antibiotic for the treatment and modifying treatment based on AST results	Review of antibiotic therapy must be done and escalated or de-escalated based on culture report	There were only 11 such cases (out of 93 patient prescriptions for urine culture) where the AST report led to the escalation or de- escalation of the therapy

ICMR: indian council of medical research.

NCDC: national centre for disease control.

### 4.8 Antimicrobial sensitivity test (AST) and antibiotic prescriptions

While a common guideline concerns the conduct of ASTs before starting clinical therapy, based on the prescriptions studied and experience of also working in the microbiology lab, that this prior testing was most often not the case. Interestingly, during the interviews, a majority of the clinicians said they would prefer to get the AST done before starting clinical therapy. One clinician said that “we have to start with the first line drug and if, in case, it comes out to be resistant, then the drug is changed”. Another clinician said that “we cannot make the patient wait, we have to give him/her something”. Another clinician said that “if we see pus in some patients then I order AST, but also start with empirical drugs”. The dental clinicians said they did not order AST because they did not see the need as patients did not have pus. We met a female child patient who was prescribed with antibiotics and also a urine culture test. We asked the patient’s mother if they had taken the medicine or not? The mother said the clinicians had asked to start the medicine after the report of culture from the lab and not before that. When the patient came to take their report, we asked if they had started the medicines, to which the mother replied she would do so only after she got the report. Given that in most cases, antibiotics were prescribed without prior AST tests, this case was relatively unusual. Our observational data showed that for skin infections, UTI, and diarrhoea, empirical drugs were started before ASTs. Also, surprisingly, we also noted that young female clinician avoided prescribing antibiotics and instead advised lifestyle and dietary changes. Preferred the AST to be done before starting any antibiotic. While based on this singular experience, we cannot make broader generalizations, our findings resonates with Liu et al. (2019), who noted that female clinicians were less likely to prescribe antibiotics as they were more comfortable in adopting the “wait and see” principle.

### 4.9 Antibiotics commonly prescribed against common symptoms

The top most prescribed antibiotics against the most commonly seen symptoms or diagnosis from the prescription data is summarized in Table 7 below:

**TABLE 7:** Most commonly prescribed antibiotics among the most common symptoms seen.

S No.	Diagnosis/Sign/Symptom	Broad categorization	Top antibiotics prescribed for it	Class of antibiotic	Broad and narrow spectrum
1	Caries	Dental condition	Ofloxacin + ornidazole	2nd generation Fluoroquinolones	Broad spectrum
			Amoxicillin	Aminopenicillins	Broad spectrum
			Amoxicillin- Metronidazole	Aminopenicillins +2nd generation Fluoroquinolones	Broad + Narrow spectrum
2	ARI	Respiratory condition	Amoxyclav	Aminopenicillins + Beta- lactamase	Broad spectrum
			Cefixime	3rd generation Cephalosporins	Broad spectrum
			Azithromycin	Macrolides	Broad spectrum
3	UTI	Urogenital condition	Cefixime	3rd generation Cephalosporins	Broad spectrum
			Nitrofurantoin	Nitrofuran	Broad spectrum
			Amoxyclav	Aminopenicillins + beta lactamase inhibitor	Broad spectrum
4	Tonsillitis	Otolaryngological condition	Amoxyclav	Aminopenicillins + beta lactamase inhibitor	Broad spectrum
			Azithromycin	1st generation Macrolides	Broad spectrum
			Cefixime	3rd generation Cephalosporins	Broad spectrum
5	Acne vulgaris	Skin condition	Azithromycin	1st generation Macrolides	Broad spectrum
			Doxycycline	1st generation Tetracycline	Broad spectrum
6	Cellulitis	Skin condition	Amoxyclav	Aminopenicillins + beta- lactamase inhibitor	Broad spectrum
			Cefixime	3rd generation Cephalosporins	Broad spectrum
7	Fever	Respiratory condition	Azithromycin	1st generation Macrolides	Broad spectrum
			Cefixime	3rd generation Cephalosporins	Broad spectrum
			Doxycycline	1st generation Tetracycline	Broad spectrum
8	Diarrhoea	Gastroenteritis	Ciprofloxacin	2nd generation Fluoroquinolones	Broad spectrum
			Ofloxacin-ornidazole	2nd generation Fluoroquinolones	Broad spectrum
9	Pneumonia	Respiratory condition	Amoxyclav	Aminopenicillins + beta lactamase inhibitor	Broad spectrum
			Azithromycin	1st generation Macrolides	Broad spectrum

We found the most common symptoms against which antibiotics were prescribed were Dental caries, respiratory conditions like fever, Pneumonia, other skin related conditions like cellulitis, Acne, UTI (Urinary Tract infections), and Tonsillitis. It was seen that the topmost antibiotic prescribed for dental caries was Ofloxacin - ornidazole followed by Amoxicillin and Amoxicillin + Metronidazole. For acute respiratory conditions (ARI), Amoxyclav was the most prescribed along with Cefixime and Azithromycin. In cases of Pneumonia, it was Amoxyclav followed by Azithromycin. For UTI, the most common antibiotic prescribed was Cefixime syrup in case of children and Nitrofurantoin in case of adults. For Tonsillitis (Otolaryngological condition) it was Amoxyclav followed by Azithromycin and Cefixime. For skin cellulitis it was Amoxyclav followed by Cefixime.

When the pharmacist was asked to comment on the prescriptions, he mentioned that “now diagnosis is mostly been written on mostly all the prescriptions since the last couple of years (after the opening of the microbiology lab in 2021)”. Earlier that was not the case, and the prescriptions did not have symptoms or diagnosis written. When we asked the pharmacist to show one slip with a diagnosis written on it, he showed one which had cough and fever as symptoms and the medicines prescribed included Azithromycin with Paracetamol for 3 days. We inferred that the pharmacist was referring to signs and symptoms as diagnosis. The different types of diagnosis (provisional/confirmatory) recorded included Respiratory infections, Tonsillitis, Acute Suppurative Otitis Media, Urinary Tract Infections), Cellulitis, Abscess and Dental caries. We found 43% of the prescriptions to be without diagnosis and to be based on signs and symptoms, such as pain in the tooth, sore throat, white discharge, pain in the abdomen, blurring vision, wound, etc. The most commonly mentioned signs and symptoms in the prescription and the supporting diagnosis are summarized in Table 8 below.

**TABLE 8:** Common signs/symptoms/diagnosis mentioned on the prescriptions.

S. No.	Sign/symptoms seen on the prescription	Presumptive diagnosis	Confirmed diagnosis
1	Pain in tooth with caries		
2	Pain in abdomen	UTI??	
3	Burning micturation and pain in abdomen		
4	Acute gastritis, Cough from 5 days with fever		A/c gastritis
5	Pain in ear with pus		ASOM
6	Tooth extracted		
7	Enlarged tonsils, fever		A/c Tonsillitis
8	fall and cellulitis on Lt lateral forehead, No Fever		Cellulitis in head

Further, we compared the most commonly prescribed antibiotics based on the quantitative analysis of the prescriptions with the interview data with clinicians (Table 9).

**TABLE 9:** Comparative analysis of antibiotics prescriptions based on quantitative data and interviews with clinicians.

S.No.	Diagnosis mentioned by clinicians requiring antibiotics prescriptions	Correct antibiotic prescription (as per graduates with an MBBS degree)	Correct antibiotic prescription (as per specialists with an MD or a higher degree)	Antibiotics prescribed in the prescriptions studied
1	RTI	Azithromycin or Amoxicillin or Doxycycline	Amoxicillin	Amoxyclav
2	UTI	Nitrofurantoin	Nitrofurantoin	Syrup Cefixime (in case of children)
				Nitrofurantoin
3	Tonsillitis	Azithromycin	-	Amoxyclav
4	Skin Cellulitis	Doxycycline or Amoxyclav	Amoxyclav	Amoxyclav
5	Pneumonia	Amoxicillin or Doxycycline or Azithromycin	Amoxyclav or Cefixime	Amoxyclav
6	Fever	Azithromycin or Doxycycline	Doxycycline	Azithromycin

When clinicians were asked when they felt the need to prescribe antibiotics, the most common responses were Upper Respiratory tract infections, Pneumonia, Acute dysentery, Tonsillitis, any bacterial infections, Urinary Tract Infections, Skin issues (Cellulitis, abscess, Furuncle, Carbuncle), Dental cases (Cellulitis, abscess, caries). When the pharmacist was probed about his knowledge of infections, he responded that “he does not know much about infections but the most commonly prescribed antibiotics for infections were Amoxyclav, Azithromycin, Cefixime, Ofloxacin oz and Doxycycline. Further, he added that Amoxyclav was mostly prescribed for dental and skin related issues. Azithromycin was used for respiratory infection or seasonal issues like tonsillitis and bronchitis. Ofloxacin OZ was also used mostly in dental OPD and Doxycycline in respiratory problems”. While Amoxicillin, Doxycycline and Azithromycin were described by the clinicians to be most effective, the prescription data showed that the most commonly prescribed antibiotics were Amoxyclav, Cefixime, Azithromycin and Ciprofloxacin. Most clinicians said they considered their own experience for prescribing antibiotics rather than the collective experience of all clinicians.

### 4.10 Following up on understanding the patients’ perspectives

Short vignettes were developed are presented based on follow up with 9 patients who had been prescribed AST and antibiotics. For issues of space, we will not present the vignettes in full, but present our interpretations from these vignettes. We noted that in nearly all cases, the patient would wait to get the AST report before commencing on the antibiotics and tended to get the dosage prescribed. In a few cases, we found patients who started to feel better after starting antibiotics, would discontinue the medicines because they felt pain in the stomach. Also, from the perspective of the clinicians, we found that most would wait for the AST reports before prescribing the antibiotics. In another case, we found an antibiotic was prescribed before an AST result was received. However, after the report was received, based on the results, the antibiotic was changed. In most cases, the diagnosis was not mentioned in the prescriptions. In another case, the patient was prescribed a 5-day antibiotics course, but after 2 days she did not feel better, and went instead to a private hospital for treatment. Here she was cured and attributed the problem to the public clinicians for not giving her proper medicines. Another patient who had pus cells but the AST showed a sterile sample, was still given antibiotics as a precautionary measure. Another female patient who was pregnant was advised by her family members not to take excessive medicines since she was pregnant. In two cases, the patient met two different OPD clinicians, and as a result came back in each case with different antibiotics being prescribed. She stopped taking the medicines.

## 5 Discussions

This study sought to understand what the patterns of prescriptions in antibiotics in a public facility in India are, and to identify the underlying institutional, cultural and social determinants driving these patterns. However, given the immense diversity in socio-cultural, economic and health status across population groups, it is difficult to attribute any single cause for the wide disparity in antimicrobial prescription rates in different regions (Llor and Bjerrum, 2014). Furthermore, information provided on the prescription prescriptions is often limited (such as the lack of information on confirmed diagnosis and prior history of prescriptions), making this analysis challenging. However, given that the ability of the healthcare system to provide the appropriate medication to the appropriate patient is one of the most crucial factors in helping the patient to achieve good health (Ahsan et al., 2016), We have developed the analysis based on the available information collected in context of a public hospital of North India.

An important finding of this study concerns the high use of broad-spectrum antibiotics. From the 681 antibiotic prescriptions studied, 50% of them included prescriptions of broad-spectrum antimicrobials such as Amoxicillin-Clavulinic acid, Cefixime and Azithromycin. This study supports study results with (Kaur et al., 2018) who found that greater than 65% of prescriptions were from broad spectrum antimicrobials such as Amoxicillin- Clavulanic acid, Ceftriaxone, Ciprofloxacin, Clindamycin and Piperacillin-tazobectum. Further, we found the average number of drugs per prescription to be 2.6, which is higher than the WHO recommendation of 1.6–1.8 drugs per encounter, since a larger set of prescribed drugs increases the risks of drug interactions (Meenakshi et al., 2022).

**FIGURE 1:**
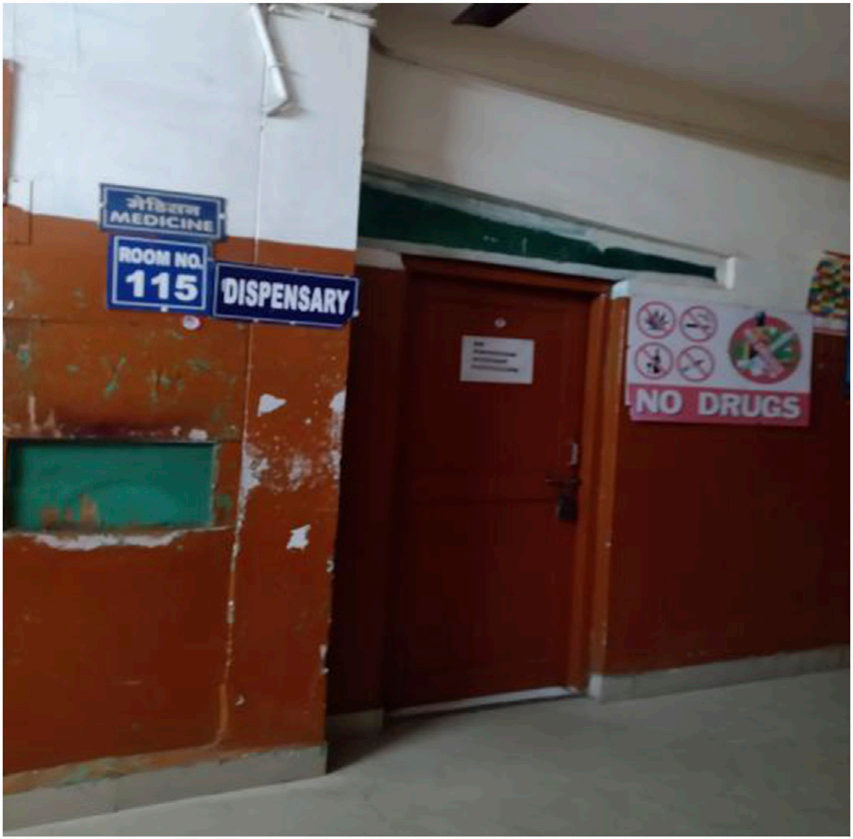
The dispensary in the hospital.

**FIGURE 2:**
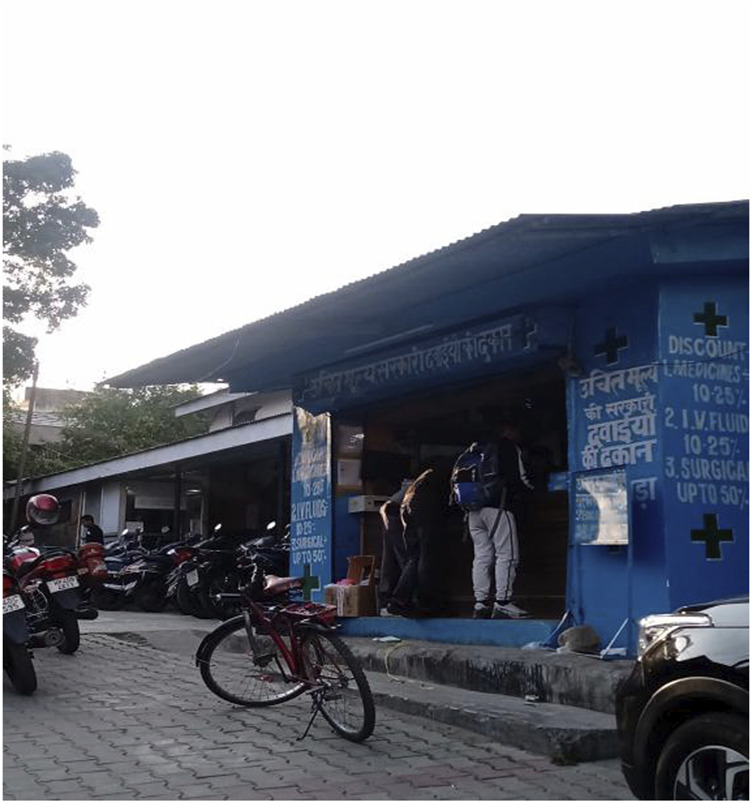
Hospital pharmacy.

**FIGURE 3:**
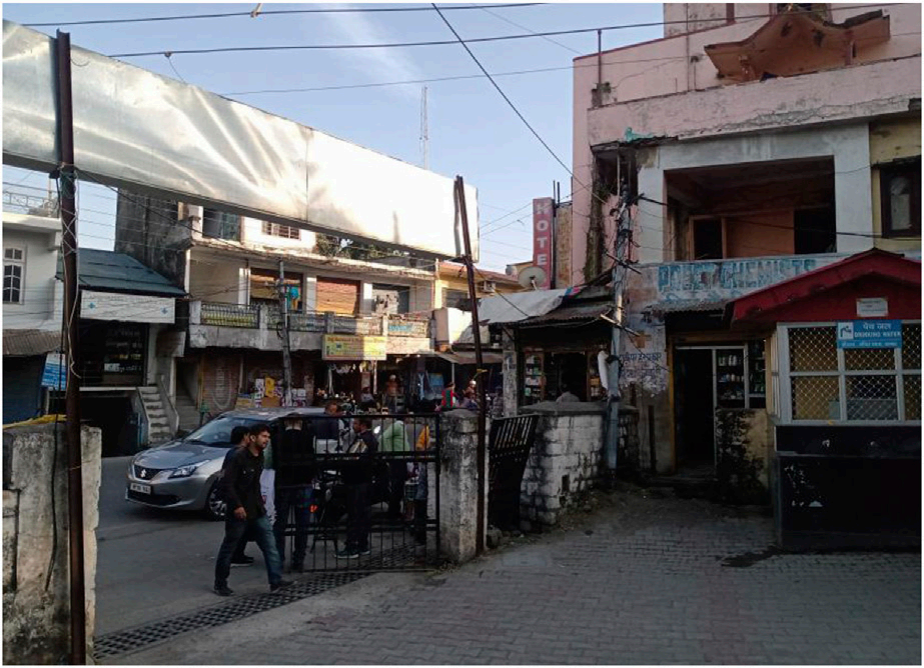
The entrance of the hospital and in house pharmacy.

From the demand side, a key social condition which may be enabling large-scale prescriptions of broad-spectrum antibiotics, concerns the next to no knowledge most patients have about antibiotics and the implications of their non-responsible use (Llor and Bjerrum, 2014). Clinicians and the hospital pharmacist rarely informed patients about the antibiotics being prescribed to them, unless, as seen in some cases, the patient had a prior acquaintance with the clinicians or the pharmacist. While pharmacists are the main source of information for the patients, they were only able to explain about drug administration and not about what is the antibiotic, why it has been prescribed and its implications. Pharmacists believed it was the duty of the clinicians to provide the patients with such information, but most clinicians said they did not have time for such counselling, and patients expressed fear of the clinicians asking more questions. The low levels of literacy and the high percentage (80% and more) of rural residents, made the patients often under-equipped to ask these questions. Patients coming from rural communities had limited literacy or knowledge regarding drugs. Further, patients themselves often demand antibiotics from the clinicians, as they felt they were not being treated appropriately without them (Sharma et al., 2021). Figures 1–3 depict the dispensary inside the hospital, the common entrance of the hospital and pharmacy and the inhouse pharmacy where the study is conducted.

On the positive side, from the total, 87.2% of the drugs prescribed were by their generic name, which is greater than another study from a tertiary care hospital in North India which found 68.5% of generic medicines (Sharma, et al., 2021). While the normative desired level on the use of generic medicine is 100% of prescriptions, this is practically impossible to achieve for various reasons, such as the perception of quality. WHO considers the prescription of generic medications as a safety measure for patients since it allows for easier information sharing, clear identification, and improved communication between medical professionals.

The study raises concerns about the overuse of antibiotics in certain OPD settings, especially dental, paediatrics and skin. In many cases, such as dental, the antibiotics may be prescribed as a precautionary measure, or due to the issue of people not being able to afford definitive dental treatment, therefore getting recurrent infections. In either case, this practice contravenes the standard guideline to prescribe antibiotics only after an AST, and only for known serious infections. We found next to no cases in which antibiotic therapy was initiated after an AST. It was interesting to note, however, during the interviews that most clinicians mentioned that they only started antibiotics to patients, after an AST was done, probably because they were aware that is the required procedure.

Further, as our vignettes point out, the patients told us that in many cases they were asked to do the AST before being prescribed antibiotics. Often the treatment was started without the AST, but after the culture reports were changed, the prescriptions were modified. We found that in a few cases, the patients stopped the medicines when they felt better, which often led to the recurrence of infections. In cases, when the medicine course was completed, the patient healed better. In a few cases, on the phone call, the husband would reply on behalf of his wife, which could not let us ascertain first-hand the outcomes.

What was reassuring was that we found that most of the drugs prescribed (98.9%) were from the Essential Drug List (EDL), which compared favorably with the claim by the clinicians expressed in the interviews that they prescribed 100% of the medicines from EDL. Other studies, for example, Meenakshi et al. (2022) have reported from a study in a teaching hospital in Southern India that 88% of the drugs prescribed were from the EDL list. However, there were differences across the OPDs, and we found 27% belonged to dental OPD, followed by 20% from pediatrics, 19.3% from general medicine, and 11% from skin and so on. Dental OPD were potentially prescribing antibiotics as a precautionary measure, which goes against the guideline of prescribing antibiotics only in the event of a confirmed infection. Such use is considered inappropriate, both therapeutically and prophylactically, and not recommended by the WHO (Sharma, 2011).

Antibiotics most preferred by physicians were found to be Doxycycline followed by Amoxyclav and Ofloxacin-Oz in case of severe infections. The most effective antibiotic noted by physicians during interviews was Amoxicillin + Metronidazole, while the prescription prescriptions showed Ofloxacin-Ornidazole followed by Amoxyclav and then Amoxicillin, to be the most prescribed. In another study, Amoxicillin was found to be the first-choice antibiotic prescribed by 39.4% of dental healthcare professionals, which is appropriate for oral infection, whereas 19.3%, 18.7% and 5.3% selected Amoxicillin/Clavulinic acid, Ofloxacin + Ornidazole and Ciprofloxacin + Inidazole, respectively, which are generally not considered necessary for oral health practice (Garg et al., 2014).

We found that 80 (11.8%) of the drugs prescribed for pediatric OPD cases were branded medicines outside the EDL. Amoxyclav and Cefixime syrup were the main antibiotics prescribed with branded names. During the interviews, a child specialist informed that there were political pressures involved in the tendering of drugs, to go for the lowest price, which potentially compromised the quality of drugs purchased by the hospital. This may be just a perception about generic drugs because of their simple packaging and tastes. A few parents told us that for themselves, they may buy drugs from the hospital pharmacy, which may be of suspect quality, but will not risk their children, even given the attraction of lower-priced generic drugs, so they go for branded more expensive drugs from private pharmacies. This behaviour can be seen as also being encouraged by the pharmaceutical nexus (Smith et al., 2021), where the medical representatives, in collusion with the clinicians and pharmacists, promote certain branded medicines. Commercial interests complemented by weak regulations, are often important drivers of the sale of sub-optimal quality branded drugs (Smith et al., 2021). An important research issue this raises is the need to study the quality of lower-priced branded drugs, whether it is a perception or reality.

When a patient visits the same physician or another, having a diagnosis written on the prescription aids in assessing the patient’s medical history, which is relevant for prescribing appropriate antibiotics, which promotes patient safety (Nash et al., 2002). In our study, we found that 48% of the prescriptions did not include a diagnosis, which when compared with a study from Ghana, shows a positive contrast which reported that 18% of prescriptions had no diagnosis written over them (Ahiabu et al., 2016). However, in an interview with a pharmacist, it was mentioned that from about the last year, there is increasing mention of diagnosis on the prescriptions as compared to the past when only signs and symptoms were mentioned, such as runny nose or sore throat, or nothing at all. In nearly no cases, we found mention of AST test results, or a change in the antibiotics taking place as a result of an AST result. However, the vignettes told a different story.

The WHO’s AWaRe classification specifies that the antibiotics consumed by the Access group should be at least 60%. As per this classification, five antibiotics (62.7%) from the Access category (Amoxyclav, Metronidazole, Doxycycline, Nitrofurantoin and Amoxicillin) and four antibiotics (37.24%) from the Watch category (Azithromycin, Ciprofloxacin, Cefixime and Ofloxacin - ornidazole) were found to be prescribed in our study (Agarwal and Chairman, 2017). In a study from Andhra Pradesh, 46.80% of antibiotics were from Access category and 53.19% were from the Watch group and their findings were in contrast to this study (Mugada et al., 2021; Kumar et al., 2022). On a positive note, no medicines were prescribed from the Reserve category.

Some recommendations for further studies include, firstly, a longitudinal study tracking prescription patterns over an extended period that can provide insights into temporal trends and variations, helping to identify emerging patterns or shifts in prescribing practices. Additionally, investigating the impact of healthcare policies, such as changes in prescription guidelines, on prescription patterns could offer valuable context and contribute to informed policy recommendations. Exploring the role of patient demographics, including age, gender, and socioeconomic factors, in prescription patterns may expose disparities and guide interventions for more equitable healthcare delivery. In our study, we analysed the quality of antibiotics based on the subjective opinion of a paediatrician (the only one at the hospital). Future studies can explore the quality of drugs at tertiary facilities with higher manpower and compare the quality among different facilities.

## 6 Conclusion

While some of our findings indicate some prudent prescriptions, such as the high percentage use of EDL listed branded drugs, other findings indicate non-prudent use, such as the rampant overuse of antibiotics more generally, and broad spectrum in particular. The absence of AST results in guiding antibiotics therapy, was another disturbing finding. However, this could be related to the limited access to diagnostics, long time taken for gaining culture results, and the general lack of knowledge and awareness amongst patients and limited time of clinicians. Some inappropriate practices were evident, such as high rates of prescriptions from dental OPD, which most probably is done as a precautionary measure. Making visible these prescription patterns to the hospital can be a motivating factor for hospital administration and practicing clinicians to understand current practices and the development of more evidence-based antibiotic stewardship guidelines.

While this analysis has focused on the sociolect-institutional determinants of prescribing, this perspective needs to be supplement with a deeper exploration of the clinical reasoning behind prescribing. This would require exploring whether clinicians/dentists genuinely think there is a bacterial infection, and how often prescribing is driven by expectations and demands of patients. It also becomes important to understand how clinicians are defining their diagnoses. For example, many diagnosis like RTI might be of viral origin and not need antibiotics. Also, the use of antibiotics for fever (of unknown cause) may often be inappropriate and would need further investigation. This could be the next enquiry stage, requiring local guidelines to help define these diagnoses further.

## Data Availability

The original contributions presented in the study are included in the article/Supplementary material, further inquiries can be directed to the corresponding author.
